# Banana Lectin: A Novel Immunomodulatory Strategy for Mitigating Inflammatory Bowel Disease

**DOI:** 10.3390/nu16111705

**Published:** 2024-05-30

**Authors:** Radmila Miljkovic, Emilija Marinkovic, Ivana Lukic, Ana Kovacevic, Zorana Lopandic, Mina Popovic, Marija Gavrovic-Jankulovic, Irma Schabussova, Aleksandra Inic-Kanada, Marijana Stojanovic

**Affiliations:** 1 Department of Research and Development, Institute of Immunology, Virology, Vaccines and Sera—Torlak, 11152 Belgrade, Serbia; rmiljkovic@torlak.rs (R.M.); emilymarinkovic84@gmail.com (E.M.); ilukic@torlak.rs (I.L.); afilipovic@torlak.rs (A.K.); 2 Institute for Chemistry in Medicine, Faculty of Medicine, University of Belgrade, 11000 Belgrade, Serbia; zorana.lopandic@med.bg.ac.rs; 3 Faculty of Ecology and Environmental Protection, University Union—Nikola Tesla, 11158 Belgrade, Serbia; minapopovic20@gmail.com; 4 Department of Biochemistry, Faculty of Chemistry, University of Belgrade, 11158 Belgrade, Serbia; mgavrov@chem.bg.ac.rs; 5 Institute of Specific Prophylaxis and Tropical Medicine, Center for Pathophysiology, Infectiology and Immunology, Medical University of Vienna, 1090 Vienna, Austria; irma.schabussova@meduniwien.ac.at; 6 Department of Molecular Biology, Institute for Biological Research “Siniša Stanković”—National Institute of the Republic of Serbia, University of Belgrade, 11108 Belgrade, Serbia

**Keywords:** banana lectin, inflammatory bowel diseases, anti-inflammatory activity, vaccination, prevention

## Abstract

Compared to the general population, patients with inflammatory bowel disease (IBD) are less likely to be vaccinated, putting them at an increased risk of vaccine-preventable illnesses. This risk is further compounded by the immunosuppressive therapies commonly used in IBD management. Therefore, developing new treatments for IBD that maintain immune function is crucial, as successful management can lead to better vaccination outcomes and overall health for these patients. Here, we investigate the potential of recombinant banana lectin (rBanLec) as a supporting therapeutic measure to improve IBD control and possibly increase vaccination rates among IBD patients. By examining the therapeutic efficacy of rBanLec in a murine model of experimental colitis, we aim to lay the foundation for its application in improving vaccination outcomes. After inducing experimental colitis in C57BL/6 and BALB/c mice with 2,4,6-trinitrobenzene sulfonic acid, we treated animals orally with varying doses of rBanLec 0.1–10 µg/mL (0.01—1 µg/dose) during the course of the disease. We assessed the severity of colitis and rBanLec’s modulation of the immune response compared to control groups. rBanLec administration resulted in an inverse dose–response reduction in colitis severity (less pronounced weight loss, less shortening of the colon) and an improved recovery profile, highlighting its therapeutic potential. Notably, rBanLec-treated mice exhibited significant modulation of the immune response, favoring anti-inflammatory pathways (primarily reduction in a local [TNFα]/[IL-10]) crucial for effective vaccination. Our findings suggest that rBanLec could mitigate the adverse effects of immunosuppressive therapy on vaccine responsiveness in IBD patients. By improving the underlying immune response, rBanLec may increase the efficacy of vaccinations, offering a dual benefit of disease management and prevention of vaccine-preventable illnesses. Further studies are required to translate these findings into clinical practice.

## 1. Introduction

Inflammatory bowel diseases (IBDs) are becoming increasingly common around the world and severely affect people’s quality of life [1]. These conditions, including Crohn’s disease and ulcerative colitis, cause gastrointestinal issues, leading to problems outside the digestive system [2]. People with IBD are more likely to experience various health complications than those without IBD [3]. Additionally, individuals with IBD are less often vaccinated than the general population, which puts them at greater risk for diseases that vaccines can prevent [4]. Given that IBD treatment typically suppresses the immune system, this elevates the risk of infections, thereby amplifying the importance of vaccinations for individuals with IBD [5].

The etiology of IBDs is complex and still needs to be fully understood. These diseases arise from the mucosal immune system’s improper reaction to the resident microbiota and other antigens available in the gut [6]. It was demonstrated that the likelihood of developing IBD is influenced by genetic predispositions and various environmental factors that an individual is exposed to throughout their life, collectively referred to as the exposome [7]. The concept of the exposome accounts for all environmental exposures a person faces, complementing the genome [8]. Among environmental factors, nutrition plays a significant role in influencing the risk and progression of IBD [9], highlighting the importance of considering both our genes and our environment in understanding the disease.

Several studies suggest that nutrition is essential to managing IBDs [10,11]. Food provides energy and supplies vital nutrients like amino acids, minerals, and vitamins for our body’s optimal metabolism. Additionally, certain food ingredients can directly influence the immune system, acting as immunomodulators, or indirectly, such as prebiotics that support gut health [12]. Given that the state of the immune system, both at the onset and throughout the progression of IBD, significantly affects the severity of the disease, it is understandable why nutrition is a critical element in managing IBD. A well-balanced diet can help mitigate disease severity and improve overall health outcomes for individuals with IBD [13].

Bananas (*Musa* sp.) are abundant in everyday nutrition, and many reports exist on their beneficial impact on the gastrointestinal tract under physiologic and specific pathogenic conditions [14]. Bananas are recommended as a part of the diet in patients suffering from IBDs as they are reported to contribute to the alleviation of disease symptoms [10,15].

Recent findings indicate that a bioactive banana’s ingredient, banana lectin (BanLec), might contribute to the observed beneficial effects. BanLec, a naturally occurring component of bananas, varies in quantity based on the fruit’s ripeness and environmental conditions [16]. BanLec, including its naturally occurring and recombinant (rBanLec) isoforms, is stable in the gastrointestinal tract and acts as an immunomodulator [17,18]. (r)BanLec is a T cell mitogen and affects the functional characteristics of T cells and macrophages [19,20,21,22,23].

rBanLec, which closely resembles the natural BanLec in structure and function, can alter the cytokine profile in the colon when applied locally [21]. It induces changes in cytokines production in a dose-dependent manner, helping to regulate the immune response’s intensity and supporting a more balanced inflammatory profile [17].

Considering the potential benefits of BanLec, we focused on examining its effects on experimental colitis outcomes when applied orally during the course of the disease. We conducted our experiments on two mouse strains possessing different genetic backgrounds, C57BL/6 (Th1-prone) and BALB/c (Th2-prone) [24], utilizing a model of colitis induced by 2,4,6-trinitrobenzene sulfonic acid (TNBS). We specifically tested the rBanLec isoform’s effectiveness as a continuous treatment strategy. The primary objective was to determine rBanLec’s potential as an adjunctive treatment for IBD. If our approach proves effective, it can advance the treatment of IBD, positively impact patient well-being, and indirectly increase vaccination uptake rates by mitigating the symptoms associated with IBD.

## 2. Materials and Methods

### 2.1. Experimental Design

For our experiments, we used ten-week-old BALB/c and C57BL/6 mice, each weighing approximately 20 g. rBanLec (GenBank accession number EU055641) used for treatments was produced and purified according to the previously described procedure [18,25] and dissolved in phosphate-buffered saline (pH = 7.4; PBS) to make solutions with rBanLec concentrations of 0.1, 1, or 10 µg/mL. Before application, all rBanLec solutions were sterilized by filtration through Ø 0.22 µm filters. Apart from a required overnight fast before receiving intrarectal treatments, animals took food and water ad libitum.

#### 2.1.1. Induction of Experimental Colitis

Experimental colitis was induced by a single-dose intrarectal administration of TNBS dissolved in 50% ethanol (25 mg/mL, 100 µL/dose; 2.5 mg TNBS/dose) on overnight fasted mice under anesthesia (10 mg/mL ketamine and 1 mg/mL xylazine in PBS, ~100 µL/mice, intraperitoneally). The day of experimental colitis induction was allotted as day 0.

#### 2.1.2. rBanLec Dosage in a Therapeutical Setting

rBanLec treatment upon the induction of experimental colitis involved daily administration, by oral route (100 µL/mice), of solutions containing 0.1, 1, and 10 µg/mL rBanLec. Thus, mice received 0.5, 5, and 50 mg rBanLec/kg BW/day.

#### 2.1.3. Treatment with rBanLec after the Induction of Colitis and Monitoring of Mice 

rBanLec therapy was initiated at the onset of the disease and was maintained consistently for a period of one week, from day 0 through day 7. Depending on the rBanLec dosage, mice of both strains were divided into three groups (n = 15 mice/group). Three groups assigned: (1) rBL0.1, (2) rBL1, and (3) rBL10 were treated with solutions (100 µL/day) containing 0.1, 1, and 10 µg/mL rBanLec, respectively. Additionally, we included two control groups to scale the effects of rBanLec against both (1) standard disease progression and (2) a non-diseased state in both strains used. The Negative Control (NC) group, comprising 15 age-matched mice, was administered a single intrarectal dose of 100 µL of 50% ethanol on day 0. This served to observe the effects of a vehicle without the active disease inducer. The Positive Control (PC) group, also with 15 age-matched mice, received a 100 µL of 25 mg/mL of TNBS dissolved in 50% ethanol intrarectally on day 0, replicating the disease conditions but without the administration of rBanLec, to establish the baseline disease response.

We monitored the body weight (BW) of the animals daily. Changes in BW, expressed as percentages, were used as a primary indicator of disease severity. From each group, five mice were randomly selected and euthanized at the peak of the disease (day 2 for C57BL/6 mice, day 3 for BALB/c mice), and another ten mice were observed until day 7, the recovery phase. Colon samples collected from the euthanized mice were prepared for tissue homogenates. Segments of colons collected from C57BL/6 mice were processed for histological analysis. Additionally, we measured and recorded the length of these colons at the peak of the disease.

### 2.2. Histochemical Analysis

Colon tissue specimens were rinsed with PBS and fixed in 4% formalin. Fixed colon samples were dehydrated in an ethyl alcohol series, cleared in xylene, and embedded in paraffin at 55 °C. Tissue sections (thickness 5 μm) were cut on a microtome (Leica RM 2155) and mounted on superfrost glass slides (Thermo Scientific, Waltham, MA, USA). Primary histological analysis was performed on sections subjected to the hematoxylin/eosin (HE) staining according to the standard procedure. HE-stained sections were analyzed using an Olympus BH2-RFL light microscope (Olympus Optica Ltd., Tokyo, Japan) with a High-Resolution Digital Camera (Color View III, Olympus Soft Imaging Solutions, Munster, Germany). An experienced pathologist who was blinded for the experimental groups performed histopathology analysis. The Mouse Colitis Histology Index (MCHI) was performed in line with the recommendations described by Koelink et al. [26].

For confocal microscopy, tissue sections were stained using an anti-mouse CD45 antibody conjugated with phycoerythrin (Biolegend, San Diego, CA, USA) and counter-stained with propidium iodide. After rinsing in PBS, the slides were mounted with a Fluorescent Mounting Medium (Dako, Glostrup, Denmark). Images were captured by an inverted confocal microscope Zeiss Laser scanning system LSM 510 Meta (Carl Zeiss, Jena, Germany) and processed with Zeiss LSM 5 Image Browser software version 4.0.

### 2.3. Analysis of T Regulatory Cells (Treg) by Flow Cytometry

Flow cytometric analysis was performed on single-cell suspensions prepared from mesenteric lymph nodes (MLN) that were isolated from C57BL/6 mice at the peak of the disease (day 2) and during the recovery (day 7). MLNs were mechanically disrupted in RPMI1640 medium supplemented with 5% FCS. The obtained cell suspension was passed through a strainer (pore size 70 µm). Cells were washed in ice-cold 2% BSA/0.01% NaN3/PBS. Staining (30 min at 4 °C in the dark) of surface markers CD4 and CD25 was performed by specific monoclonal antibodies conjugated with phycoerythrin and allophycocyanin, respectively (Biolegend), appropriately diluted in 2% BSA/0.01% NaN3/PBS. After washing, cells were subjected to fixation and permeabilization by Foxp3/transcription Factor Staining Buffer Set (eBioscience, Carlsbad, CA, USA) according to the manufacturer’s instructions. Foxp3 was stained by a specific monoclonal antibody conjugated with fluorescein isothiocyanate (Biolegend). Cells were analyzed by BD FACSVerse™ flow cytometer (BD Bioscience, San Jose, CA, USA) equipped by FACSuite Software v1.0.5.3841.

### 2.4. Preparation of Colon Homogenates

The colon was rinsed with a cold PBS, weighted, and embedded in the buffer for homogenization (cold PBS supplemented with 1 mM EDTA, 0.1% NP-40, 1% Triton X-100, and 1% cocktail of protease inhibitors (Serva, Heidelberg, Germany). Upon homogenization (Janke & Kunkel, Staufen, Germany), samples were centrifuged (18,000× *g*, 10 min, 4 °C; Centrifuge 3K-18, Sigma, Osterode, Germany), and supernatants were collected for further analyses. The total protein concentration in supernatants was determined following Bradford’s method. Supernatants were stored at −80 °C until further analysis.

### 2.5. Evaluation of Local NO Production

NO production was evaluated by quantification of its stable end product (nitrite) using a Griess reagent. An equal volume of Griess reagent (1% sulphanilamide, 0.1% N-(1-naphthyl)-ethylenediamine dihydrochloride in 5% phosphoric acid) and fresh supernatants of homogenized colonic samples (100 µ/well) was added to the 96-well plate and incubated for 10 min at room temperature (RT). After incubation, the absorbances were measured at 545 nm, and concentrations were calculated from a linear standard curve prepared using sodium nitrite.

### 2.6. Evaluation of Local Myeloperoxidase (MPO) Activity

The evaluation of MPO activity was based on MPO’s oxidation of o-phenylenediamine (Sigma, Saint Louis, MO, USA). Supernatants of colonic homogenates were adjusted to the same protein concentration (1 mg/mL) before analysis. Equal volumes (75 µL) of supernatant and substrate solution (1 mg/mL phenylenediamine, 0.01% hydrogen peroxide, 50 mM citric acid, pH = 5) were mixed in a 96-well plate (in a total of 50 µL/well). After incubation for 10 min at RT, the reaction was terminated by adding 50 µL/well of 1M sulphuric acid. The absorbance was measured at 492 nm and 620 nm (A492/620).

### 2.7. Analysis of IL-10 and TNFα Production

Concentrations of IL-10 and TNFα were measured by a sandwich ELISA in the supernatants of the colon homogenates collected at the peak of TNBS-induced colitis. Capturing antibody against either IL-10 (eBioscience; 2 µg/mL in PBS) or TNFα (Biolegend; 3 µg/mL in PBS), respectively, was adsorbed (50 µL/well; overnight at 4 °C) onto a microtiter plate (MaxiSorp; Nunc, Roskilde, Denmark). Upon blocking with 1% *w*/*v* BSA/PBS, supernatants and commercially available standards (1:2 serial dilutions prepared in 1% *w*/*v* BSA/PBS) were added to the wells (50 µL/well; 1 h at RT). IL-10 and TNFα were detected with biotin-labeled anti-mouse IL-10 (eBioscience; 1 µg/mL in 1% *w*/*v* BSA/PBS) and anti-mouse TNFα (Biolegend; 0.5 µg/mL in 1% *w*/*v* BSA/PBS), respectively (50 µL/well; 1 h at RT). Blocking and each subsequent step was followed by a washing step with a 0.05% *v*/*v* Tween 20/PBS (4 × 200 µL/well). ExtrAvidin-peroxidase (Sigma, USA)/OPD system was used for the visualization, and absorbance was read at 492 nm and 620 nm (Multiscan Ascent, Labsystems, Helsinki, Finland).

### 2.8. Statistical Analysis

The statistical significance of differences in parameter values measured at specified time-point was assessed using a One-way ANOVA, followed by Bonferroni’s multiple comparison test. Additionally, Pearson’s bivariate correlation analysis was employed to calculate Pearson’s correlation coefficient (Pcc), which reflects the relationship between specific variables. A probability (*p*) value of 0.05 was considered the threshold for significance for all analyses.

## 3. Results

### 3.1. Oral Application of rBanLec throughout TNBS-Induced Colitis Reduces the Disease Severity in an Inverse Dose-Dependent Manner

**Figure 1:**
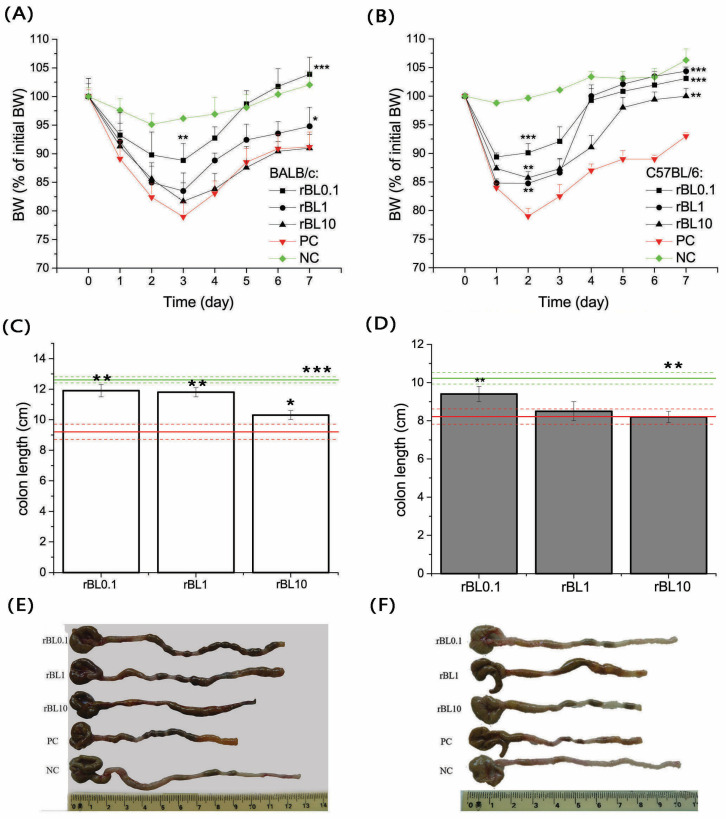
Changes in the body weight (BW) during TNBS-induced colitis and colon length at the peak of disease in mice treated with rBanLec upon the induction of experimental colitis. Changes in the BW (**A**,**B**) and colon length (**C**–**F**) due to induction of experimental colitis and rBanLec treatments were recorded for BALB/c (**A**,**C**,**E**) and C57BL/6 (**B**,**D**,**F**) mice. Values recorded for corresponding PC (red lines) and NC (green lines) groups are indicated on the plots. BW loss of mice subjected to the specific treatment (see Section 2.1.2) was recorded daily and expressed as a percentage of BW recorded on day 0. Mean BW loss ± SEM at a specified day upon the induction of the disease (n = 15 until peak of the disease, n = 10 after peak of the disease) is presented on the plot (**A**,**B**). Colon lengths were recorded at the peak of the disease (day 3 for BALB/c and day 2 for C57BL/6) and presented as mean colon length ± SEM for each group (BALB/c—white bars (**C**), C57BL/6—dark gray bars (**D**)) along with photos of representative samples (**E**,**F**). Statistical significance of differences in the BW changes and colon length compared to the corresponding PC group is evaluated by One-way ANOVA followed by Bonferroni’s multiple comparison tests (* *p* ˂ 0.05, ** *p* ˂ 0.005, *** *p* ˂ 0.001).

Induction of colitis in the experimental setup led to marked weight loss and a decrease in colon length at the disease’s peak in both BALB/c (Figure 1A) and C57BL/6 (Figure 1B) mouse strains. Notably, the weight loss in the PC group at the peak of the disease was statistically significant when compared to the corresponding NC group, with a *p*-value less than 0.001 for both strains. Similarly, a significant shortening of colon length was observed in the PC group compared to the NC group, with a *p*-value less than 0.005 and 0.001 for C57BL/6 and BALB/c mice, respectively (Figure 1C,D). The follow-up in the BW fluctuations in C57BL/6 and BALB/c mice orally treated (daily) with rBanLec upon the induction of colitis showed that the application of rBanLec in doses of 0.1 µg/mL exerts the most pronounced benefits (Figure 1A,B) in both strains.

Considering the weight loss at the peak of the disease, a significant beneficial effect in BALB/c was recorded only in mice receiving rBanLec in a concentration of 0.1 µg/mL (*p* < 0.005; Figure 1A). However, a reduction in colon length recorded at the peak of the disease implied the beneficial effects in rBL1 and rBL10 groups as well (compared to the PC group: *p* ˂ 0.005 for rBL0.1 and rBL1, *p* ˂ 0.05 for rBL10; Figure 1C,E).

In C57BL/6 mice, the beneficial effect at the peak of the disease was recorded in all rBanLec-treated groups (compared to the PC group: *p* ˂ 0.001 for rBL0.1, *p* ˂ 0.05 for rBL1 and rBL10; Figure 1B). In addition, a reduction in colon length at the peak of the disease was the lowest in the rBL0.1 group (*p* ˂ 0.005 compared to PC and rBL10, *p* ˂ 0.05 compared to rBL1; Figure 1D,F).

Treatment with rBanLec demonstrated a beneficial effect on the progression of colitis, even when administered in higher doses. This positive influence is reflected in a more robust weight recovery phase. Compared to the PC group, BALB/c mice treated with lower doses of rBanLec (rBL0.1) showed a statistically significant improvement in weight gain, with a *p*-value less than 0.001. A trend toward improving the recovery phase has been marked with BALB/c mice treated with a higher dose of rBanLec (PC vs. rBL1 *p* ˂ 0.05), as depicted in Figure 1A. In C57BL/6 groups, both doses, rBL0.1 and rBL1, resulted in a highly significant weight gain compared to the PC group, with *p*-values less than 0.001, and the highest dose (rBL10) also showed significant weight gain, with a *p*-value less than 0.005, as illustrated in Figure 1B.

### 3.2. Treatment with rBanLec Reduces Infiltration of Immune Cells in the Colon at the Peak of the Disease

Histological analyses and analyses of draining MLN were performed at the peak of the disease only for C57BL/6 mice as they are considered more susceptible to organ-specific Th1-mediated immunopathology [27,28,29,30].

**Figure 2:**
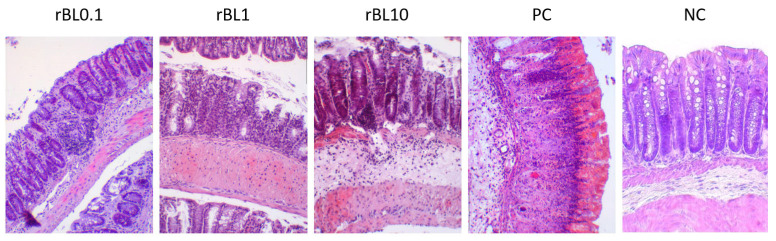
Transversal sections of the colon at the peak of TNBS-induced colitis in C57BL/6 mice treated with rBanLec throughout the disease duration. Colons of C57BL/6 mice subjected to the specific treatment (see Section 2.1.2) were collected at the peak of TNBS-induced colitis (day 2) and recovery (day 7). Tissue sections (thickness 5 µm) were stained with hematoxylin/eosin and examined (magnification 10×) using the Olympus BH2-RFL light microscope (Olympus Optica Ltd., Tokyo, Japan) equipped with High-Resolution Digital Camera (Color View III, Olympus Soft Imaging Solutions, Munster, Germany). Representative photos are presented.

Histological examination of the colon tissue from C57BL/6 mice at the peak of the disease, as shown in Figure 2, indicates that the mice treated with rBanLec retained a more intact colonic architecture and exhibited less infiltration by immune cells (identified as CD45+ cells, Appendix A) when compared to the mice in the PC group.

Histochemical analysis of colon samples collected at the peak of the disease (Figure 2) revealed that the massiveness of cell infiltrates and the extent of crypts deformation positively correlate to the disease severity assessed by weight loss (Figure 1B). Analysis of PC sections collected at the peak of the disease revealed pronounced impairment of the architecture of colonic tissue associated with the intensive destruction of Goblet cells and scattering of infiltrates of immune cells along all tissue layers, yet mostly in lamina propria. In addition, deformed crypts, impregnated with severe hemorrhagic discharge, were marked in PC colonic sections. Patchy crypt degeneration and loss of Goblet cells were also marked in tissue sections of rBanLec-treated mice, and they were the lowest in the rBL0.1 group. The hypertrophy of lamina muscularis externa as a response to the inflammation was observed in the rBL1 and rBL10 groups. Infiltrations of the immune cells were marked in all sections of rBanLec-treated mice, but in rBL10 mice, they were the most pronounced and associated with hemorrhagic zones in some places. Taking into account the parameters identified by Koelink et al. [26] as the best predictors of disease severity (Goblet cell loss, crypt density, crypt hyperplasia, and submucosal infiltrates), it can be concluded that the mildest disease was developed in rBL0.1 (MCHI = 9). In contrast, disease severity in rBL10 was comparable to the PC with an MCHI = 18 for both rBL10 and PC (details provided in Appendix B).

**Figure 3:**
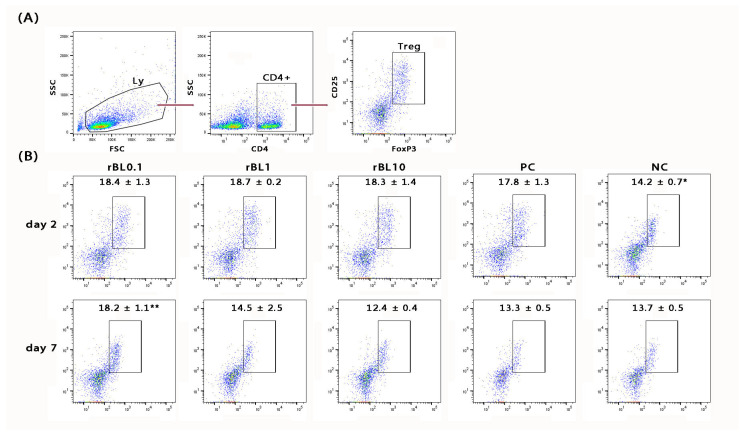
Changes in the percentage of Treg in MLNs of C57BL/6 mice that were subjected to the induction of experimental colitis by TNBS and treatment with rBanLec throughout the disease duration. Treg in MLN was measured by flow cytometry, being identified as CD4+CD25+Foxp3+ (**A**). The percentage of Treg in MLN of rBanLec-treated (rBL0.1, rBL1, rBL10), as well as control (PC, NC) mice, was determined at the peak of the TNBS-induced colitis (day 2) and recovery (day 7) (**B**). The mean percentage of Treg within CD4+ lymphocytes ± SEM per group is indicated on the plot. Statistical significance of the differences in comparison to the PC is evaluated with One-way ANOVA followed by Bonferroni’s multiple comparison test (* *p* < 0.05, ** *p* < 0.005).

In addition to histological analysis of the colon, the MLNs draining the colonic mucosa of C57BL/6 mice were analyzed for the presence of Treg (identified as CD4+CD25+Foxp3+; Figure 3A) at the peak of the disease and during the recovery phase. Flow cytometric analysis revealed a general decrease in the frequency of CD4+ cells within the MLN lymphocytic pool at the peak of the disease (rBL0.1 29.9 ± 1.8%, rBL1 30.2 ± 1.1%, rBL10 31.2 ± 0.3%, PC 33.9 ± 2.8%) compared to NC (37.4 ± 0.4%). However, irrespective of the rBanLec treatment, the pool of CD4+ lymphocytes within MLNs collected from C57BL/6 mice subjected to the induction of experimental colitis showed a higher percentage of Treg at the peak of the disease compared to NC (*p* ˂ 0.05). There were no statistically significant differences among percentages recorded for PC and rBanLec-treated groups at this time-point. Furthermore, a trend toward increased Treg abundance within the overall lymphocytic pool in the MLNs was noted despite the contraction of the CD4+ lymphocytic pool at the peak of the disease (% of Treg in lymphocytes: NC 5.4 ± 0.3%, PC 6.0 ± 0.2%, rBL0.1 5.5 ± 0.6%, rBL1 5.6 ± 0.3%, rBL10 5.7 ± 0.5%). During the recovery phase (day 7), regeneration of the CD4+ pool within the lymphocytic pool in the MLNs was observed (rBL0.1 33.2 ± 1.2%, rBL1 34.8 ± 3.2%, rBL10 39.8 ± 1.9%, PC 37.1 ± 0.9% vs. NC 37.1 ± 0.8%). In addition, the percentage of Treg in the CD4+ lymphocytes of the rBL0.1 MLNs was significantly higher compared to the MLNs of rBL10 (*p* ˂ 0.05) as well as PC (*p* ˂ 0.005) group (Figure 3B). A significantly higher percentage of Treg in rBL0.1 compared to PC (*p* ˂ 0.05) as well as NC (*p* ˂ 0.05) was retained at the level of the whole lymphocytic pool in MLNs (% of Treg in lymphocytes: NC 5.1 ± 0.2%, PC 4.9 ± 0.2%, rBL0.1 6.0 ± 0.2%, rBL1 5.1 ± 0.9%, rBL10 4.9 ± 0.1%).

### 3.3. Treatment with rBanLec Is Associated with Reductions in Local MPO Activity and NO Production at the Peak of the Disease

**Figure 4:**
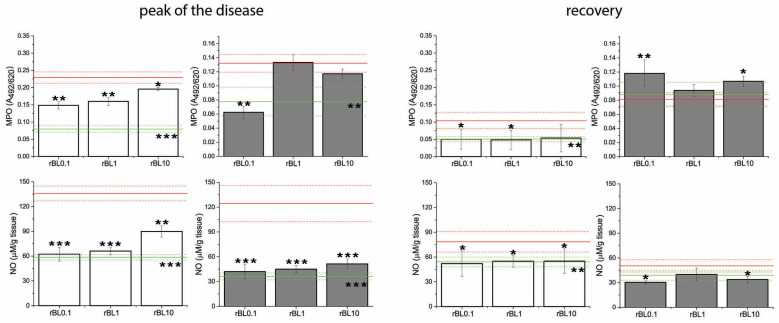
MPO activity and NO production in the colon of mice subjected to the induction of experimental colitis by TNBS and the treatment with rBanLec throughout the disease duration. MPO activity and NO production were evaluated in colon samples collected from BALB/c (white bars) and C57BL/6 (dark gray bars) at the peak of the disease (day 3 for BALB/c and day 2 for C57BL/6 mice) and the recovery (day 7). Results are presented as mean value ± SEM for each group at the indicated time-point. Red and green lines indicate values recorded in corresponding PC and NC groups (mean value—solid line, mean value ± SEM—dotted lines). Statistical significance of differences in the MPO activity and NO production compared to the corresponding PC group is evaluated by One-way ANOVA followed by Bonferroni’s multiple comparison test (* *p* < 0.05, ** *p* ˂ 0.005, *** *p* ˂ 0.001).

Measurements of local MPO activity and NO production, which are often elevated in inflammation, implied the inverse dose-dependent anti-inflammatory impact of rBanLec treatment (Figure 4). Indeed, compared to the basal level recorded in the corresponding NC, those parameters were significantly elevated in PC at the peak of the disease in both strains (BALB/c: *p* < 0.001 for both MPO activity and NO production; C57BL/6: *p* < 0.005 for MPO activity and *p* < 0.001 for NO production). Although some strain-dependent differences were marked, a low rBanLec dose (0.01 µg/mL, 100 µL per day) exerted a more substantial beneficial impact than the highest one (10 µg/mL, 100 µL per day). In BALB/c mice, both MPO activity and NO production at the peak of the disease were significantly lower in all rBanLec-treated groups compared to PC (MPO activity: *p* < 0.005 for rBL0.1 and rBL1, and *p* < 0.05 for rBL10; NO production: *p* < 0.001 for rBL0.1 and rBL1, *p* < 0.005 for rBL10). In C57BL/6 mice, rBanLec treatment significantly reduced MPO activity only in the rBL0.1 group (*p* ˂ 0.005), while NO production was significantly reduced in all rBanLec-treated groups (*p* ˂ 0.001 for rBL0.1, rBL1 and rBL10 groups) compared to PC.

Recovery from the disease for all mice subjected to the induction of experimental colitis was associated with a decline in local NO production and MPO activity compared to the peak of the disease. MPO activity and NO production recorded in recovered PC C57BL/6 mice reached levels comparable to the ones recorded in the NC, while in recovered PC of BALB/c mice, they remained higher than basal levels (PC vs. NC *p* ˂ 0.005 for both parameters). In rBanLec-treated groups of BALB/c mice, MPO activity and NO production were lower than in corresponding PC (MPO activity: *p* ˂ 0.05 for rBL0.1 and rBL1, NO production: *p* < 0.05 for rBL0.1, rBL1, and rBL10), reaching the level recorded in BALB/c NC group. The same trend was marked for NO production in recovered C57BL/6 mice (compared to PC *p* < 0.05 for rBL0.1 and rBL10). In rBL1, rBL10, and PC groups of C57BL/6 mice, local MPO activity at recovery was lower compared to the peak of the disease, reaching a level comparable to NC. Among C57BL/6 mice subjected to the induction of experimental colitis, the highest MPO activity in the recovery was recorded in the rBL0.1 group. Even more, it was higher than at the peak of the disease (*p* < 0.005).

### 3.4. Treatment with rBanLec Modulates the Local [TNFα]/[IL-10] Ratio at the Peak of the Disease

**Figure 5:**
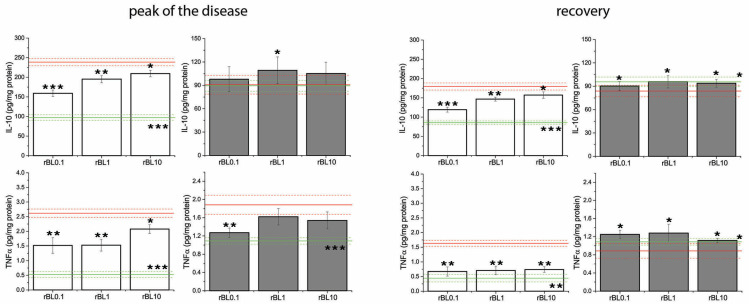
Concentrations of IL-10 and TNFα in the colon of mice subjected to the induction of experimental colitis by TNBS and the treatment with rBanLec throughout the disease duration. Concentrations of TNFα and IL-10 were measured in homogenates of colon samples collected from BALB/c (white bars) and C57BL/6 (dark gray bars) at the peak of the disease (day 3 for BALB/c and day 2 for C57BL/6 mice) and the recovery (day 7). Results are presented as mean value ± SEM for each group at the indicated time-point. Red and green lines indicate values recorded in corresponding PC and NC groups (mean value—solid line, mean value ± SEM—dotted lines). Statistical significance of differences in the local concentrations of TNFα and IL-10 compared to the corresponding PC group is evaluated by One-way ANOVA followed by Bonferroni’s multiple comparison test (* *p* ˂ 0.05, ** *p* ˂ 0.005, *** *p* ˂ 0.001).

The measurement of local concentrations of the TNFα and IL-10 cytokines with a pronounced pro-inflammatory and anti-inflammatory impact, respectively, confirmed that an increase in the contribution of the anti-inflammatory immune response accompanied the reduction in disease severity at the peak of the disease. Irrespective of the rBanLec treatment, induction of experimental colitis in both strains was associated with a simultaneous rise in local production of both TNFα and IL-10 (Figure 5).

Among groups subjected to the induction of experimental colitis, the most intensive increase in local concentrations of TNFα at the peak of the disease was recorded in PC groups for both strains. In BALB/c mice, local concentrations of TNFα and IL-10 at the peak of the disease were significantly higher in PC (*p* ˂ 0.001 for both cytokines) as well as rBanLec-treated mice (IL-10: *p* ˂ 0.005 for rBL0.1, *p* ˂ 0.001 for rBL1 and rBL10; TNFα: *p* ˂ 0.005 for rBL0.1 and rBL1, *p* ˂ 0.001 for rBL10) than basal concentrations recorded in NC. A significant increase in local TNFα concentration at the peak of the disease was recorded in C57BL/6 mice as well (compared to NC: *p* ˂ 0.05 for rBL0.1, *p* ˂ 0.005 for rBL10, *p* ˂ 0.001 for rBL1 and PC). The increase in local IL-10 concentration at the peak of the disease was not pronounced in either group of C57BL/6 mice subjected to the induction of experimental colitis. However, it was significantly higher in rBanLec-treated groups than in PC (*p* ˂ 0.05 in rBL1).

**Figure 6:**
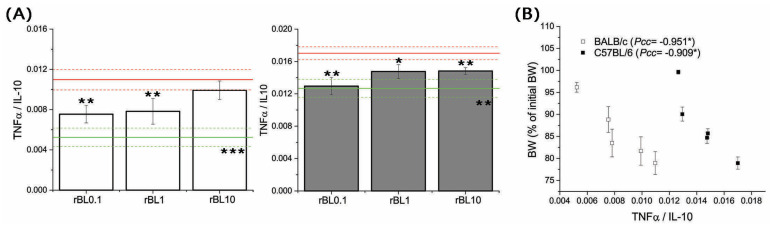
Local [TNFα]/[IL-10] and its correlation with the severity of TNBS-induced colitis at the peak of the disease. [TNFα]/[IL-10] (**A**) was determined in homogenates of colon samples collected from BALB/c (white bars) and C57BL/6 (dark gray bars) at the peak of the disease (day 3 for BALB/c and day 2 for C57BL/6 mice). Results are presented as mean [TNFα]/[IL-10] ± SEM for each group. Red and green lines indicate values calculated for corresponding PC and NC groups (mean value—solid line, mean value ± SEM—dotted lines). Statistical significance of differences in the [TNFα]/[IL-10] compared to the corresponding PC group is evaluated by One-way ANOVA followed by Bonferroni’s multiple comparison test (* *p* ˂ 0.05, ** *p* ˂ 0.005, *** *p* ˂ 0.001). Analysis of the correlation between mean BW and local [TNFα]/[IL-10] calculated for each experimental group at the peak of disease (**B**) was performed by Pearson’s bivariate correlation analysis; Pcc—Pearson’s correlation coefficient, * *p* ˂ 0.05.

In addition, analysis of local [TNFα]/[IL-10] ratio at the peak of the disease revealed it is decreasing due to rBanLec treatment (Figure 6A), most intensively in rBL0.1 groups of both strains (rBL0.1 compared to corresponding PC: *p* ˂ 0.005 for both strains). Correlation analysis summarizing presented data revealed a significant correlation between the severity of pathology (estimated via BW loss) and a local [TNFα]/[IL-10] ratio at the peak of disease (Pcc = −0.951, *p* ˂ 0.05 for BALB/c, Pcc = −0.909, *p* ˂ 0.05 for C57BL/6; Figure 6B).

The recovery was associated with a sharp drop in local TNFα concentration (Figure 5), which in all groups subjected to the induction of experimental colitis except the PC group of BALB/c mice (PC vs. NC *p* ˂ 0.005 for BALB/c) reached a level comparable to the one recorded in corresponding NC. Contrary to the groups of C57BL/6 mice subjected to the induction of experimental colitis where local IL-10 levels were similar to NC (Figure 5), local IL-10 levels remained elevated in recovered BALB/c mice (compared to NC: *p* ˂ 0.005 for rBL0.1, *p* ˂ 0.001 for rBL1, rBL10, and PC).

## 4. Discussion

The presented results show that rBanLec significantly modulates the local immune response in the colon by reducing the severity and supporting the recovery from experimental colitis.

Intrarectal TNBS administration to mice induces transmural colitis mainly driven by a Th1-mediated immune response and characterized by local infiltration of the lamina propria with T cells, neutrophils, and macrophages as well as some clinical features of Crohn’s disease (severe diarrhea, weight loss, and rectal prolapse) [31]. Hence, it is widely used to study immunologic aspects relevant to Crohn’s disease. Testing of rBanLec therapeutic potential was performed on two mouse strains, BALB/c and C57BL/6, which are highly different concerning susceptibility to inflammatory-mediated diseases, including IBDs [32]. Experimental colitis could be induced in both strains, although there are conflicting data on strain-specific susceptibility toward induction of experimental colitis depending on the applied method. This could be attributed to the specific characteristics of gut microbiota and differences in gut-associated lymphoid tissue (GALT)-related immunological profiles encompassing the proportion of T cell populations in GALT and draining lymph nodes as well as the specific kinetics of expression of various mediators during the establishment of an immune response [24]. Those factors result in generally stronger inflammatory responses to the same stimuli in C57BL/6 compared to BALB/c mice. Hence, BALB/c mice are considered to be more resistant to organ-specific Th1-mediated immunopathology than C57BL/6 mice [27,28,29,30]. Furthermore, as demonstrated for peritoneal macrophages, the response to rBanLec stimulation of C57BL/6 and BALB/c mice is not the same [23].

Although the therapeutic administration of rBanLec in a tested dose range (0.01–1 µg/dose) could not fully prevent the development of experimental colitis, important beneficial effects were observed. The efficacy of rBanLec as a treatment for experimental colitis notably showed an inverse dose-dependent relationship. Post-induction therapeutic application of rBanLec revealed that its advantageous effects were more evident with lower doses. Following colitis induction, a daily regimen of rBanLec at a minimal dose (0.01 µg/dose) led to substantial mitigation of disease severity in both mouse strains. However, even in higher doses, rBanLec positively impacted the overall course of the disease.

The positive effects of rBanLec were evident in the moderated weight loss and the reduced shortening of the colon during the most intense phase of the disease. These observable changes could be attributed to decreased cellular infiltration in the colonic tissue and the initiation of an anti-inflammatory immune response following the onset of colitis. The suppression of inflammation in the colon was further indicated by increased production of the regulatory cytokine IL-10 and a diminished pro-inflammatory response, as evidenced by the lowered ratio of TNFα to IL-10, decreased NO production, and reduced MPO activity.

In our study, we posit that the upregulation of pro-inflammatory cytokines may necessitate a higher level of rBanLec stimulation compared to what is required for augmenting the production of regulatory cytokines. The observed ratio of local IL-10 to TNFα concentrations at the apex of the disease suggests that lower doses of rBanLec tip the balance in favor of an anti-inflammatory environment (evidenced by higher IL-10 levels) rather than an inflammatory one (indicated by lower TNFα levels). The results point to strain-specific pathways for the observed reduction in the local [TNFα]/[IL-10] ratio in response to the rBanLec treatment. Specifically, this was attributed to decreased TNFα production in C57BL/6 mice, while a notable increase in IL-10 production was instrumental for the BALB/c mice. A similar observation related to strain-specific kinetic of IL-10 expression in a mouse model of DSS-induced colitis was reported by Mukhopadhyay et al. [32]. Further, different MPO kinetics during recovery of C57BL/6 and BALB/c mice imply additional strain-specific differences in mechanisms underlining recovery. It implies the important role of neutrophils in the resolution of inflammation in C57BL/6 mice [33]. Besides alleviating local inflammation, it might as well be that rBanLec, as a TLR-2 agonist [23], supported the restoration of compromised epithelial integrity [34].

The immune system’s attempts to regulate colonic inflammation are reflected in the augmented differentiation of Tregs within the MLNs. At the climax of the disease, the increase in Treg proportions was consistent across all diseased mice, whether treated with rBanLec or not, indicating a systemic attempt to limit inflammation. Notably, the elevated Treg levels were sustained during the recovery phase exclusively in the cohort treated with a low dose of rBanLec (rBL0.1). This could suggest a prolonged and enhanced capacity of the immune system to forestall further intense inflammatory responses.

Our findings reveal that both rBanLec and BanLec-containing foods hold significant promise for treating IBDs. We have demonstrated that rBanLec can positively influence the local environment in the colon, promoting regulatory and anti-inflammatory responses. However, the finding that the impact of rBanLec is highly dose-dependent highlights the importance of careful consideration in formulating rBanLec-based treatments. Daily treatment with 0.01 µg rBanLec (i.e., 0.5 mg rBanLec/kg BW/day) was the most efficient IBD attenuation in both mice strains. That dosage extrapolated to humans would be ~0.04 mg rBanLec/kg BW/day [35]. That therapeutic dosage for a human weighing 70 kg (~2.8 mg BanLec) could be found in one banana (average BanLec content is ~4 mg/100 g banana pulp [36]; a medium-sized banana contains ~120 g of banana pulp [37]. As we observe certain differences in different genetic backgrounds, BanLec could be a part of a personalized application strategy in the future.

Elucidating the underlying processes by which rBanLec confers its therapeutic benefits is pivotal to developing a potent and secure treatment regimen for IBD using BanLec.

## 5. Conclusions

Should our results be confirmed through further research, it could offer a new avenue for the treatment and management of IBD patients and increase the likelihood of these patients vaccinating more frequently. This is particularly significant considering the compromised immune systems of IBD patients and their increased risk for vaccine-preventable diseases. In conclusion, our study highlights the potential of BanLec as a key component in advancing IBD treatment and improving patient outcomes.

## Figures and Tables

**Figure A1:**
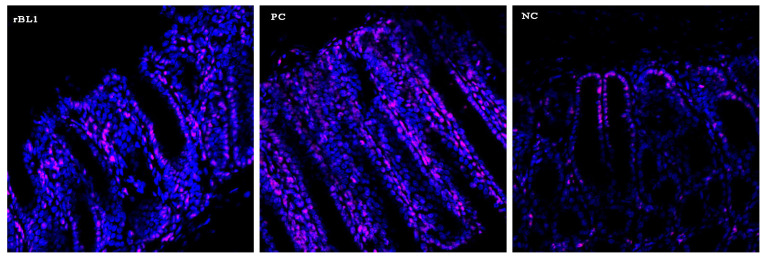
Analysis of infiltration of immune cells at the peak of TNBS-induced colitis by confocal microscopy. Sections of colons collected from NC, PC, and rBL10 groups of C57BL/6 mice were stained with anti-mouse CD45-PE antibody (pink) and counter-stained by PI (blue). Representative samples are presented. Confocal microscopy (LSM 510 Meta).

## Data Availability

The original contributions presented in this study are included in the article. Further inquiries can be directed to the corresponding author.

## Appendix B

**Table A1:** MCHI calculated for colons of C57BL/6 mice at the peak of the disease.

	Goblet Cell Loss	Crypt Density *	Crypt Hyperplasia *	Submucosal Infiltrate **	Total Score
rBL0.1	1	1	0	2	9
rBL1	2	2	0	2	12
rBL10	3	2	1	3	18
PC	3	2	1	3	18
NC	0	0	0	0	0

* multiply by 2, ** multiply by 3.
